# The Swedish Medical Products Agency’s rules of procedure

**DOI:** 10.1186/s12961-018-0372-3

**Published:** 2018-11-15

**Authors:** Catarina Andersson Forsman

**Affiliations:** Swedish Medical Products Agency, SE -751 03 Uppsala, Sweden

The Swedish Medical Products Agency (MPA) is the Swedish national authority responsible for regulation and surveillance of the development, manufacturing and marketing of medicinal products. Relenza, a neuraminidase inhibitor used in the treatment of influenza, has been evaluated by both the Swedish MPA and the United States Food and Drug Administration.

In a paper by Mulinari et al. [1], the divergent conclusions reached by the authorities are discussed. The purpose of this letter is not to discuss the conclusions drawn by Mulinari et al. [1], but to comment on the way they refer to an assessor at the MPA. By naming a specific assessor at the MPA, the reader is given the false impression that a single employee is solely responsible for the benefit–risk evaluation of a drug. We want to emphasize that all assessments at the MPA are the work of a team of assessors with complementary competencies, including a comprehensive standardized quality assessment procedure for each decision. Hence, it is the MPA, as a national regulatory agency, that is responsible for any opinion or decision. Besides giving a misleading description of regulatory procedures, the publication of an individual assessor’s name adds no scientific value to the paper and could therefore have been omitted.
